# What proportion of AQP4-IgG-negative NMO spectrum disorder patients are MOG-IgG positive? A cross sectional study of 132 patients

**DOI:** 10.1007/s00415-017-8596-7

**Published:** 2017-08-24

**Authors:** Shahd H. M. Hamid, Daniel Whittam, Kerry Mutch, Samantha Linaker, Tom Solomon, Kumar Das, Maneesh Bhojak, Anu Jacob

**Affiliations:** 1 The Walton Centre NHS Foundation Trust, Liverpool, L9 7LJ UK; 2 Institute of Infectious Disease and Global Health, University of Liverpool, The Walton Centre NHS Foundation Trust, Liverpool, UK

**Keywords:** Neuromyelitis optica, Aquaporin-4 antibodies, Myelin oligodendrocytes glycoprotein

## Abstract

Antibodies to myelin oligodendrocyte glycoprotein (MOG-IgG) have been described in patients with neuromyelitis optica spectrum disorders (NMOSD) without aquaporin-4 antibodies (AQP4-IgG). We aimed to identify the proportion of AQP4-IgG-negative NMOSD patients who are seropositive for MOG-IgG. In a cross sectional study, we reviewed all patients seen in the National NMO clinic over the last 4 years (after the availability of MOG-IgG testing), including clinical information, MRI, and antibody tests. 261 unique patients were identified. 132 cases satisfied the 2015 NMOSD diagnostic criteria. Of these, 96 (73%) were AQP4-IgG positive and 36 (27%) were AQP4-IgG negative. These 36 patients were tested for MOG-IgG and 15/36 (42%) tested positive. 20% (25/125) of the patients who did not satisfy NMOSD criteria had MOG-IgG. Approximately half of seronegative NMOSD is MOG-Ig seropositive and one in five of non-NMOSD/non-MS demyelination is MOG-IgG positive. Since MOG-associated demyelinating disease is likely different from AQP4-IgG disease in terms of underlying disease mechanisms, relapse risk and possibly treatment, testing for MOG-IgG in patients with AQP4-IgG-negative NMOSD and other non-MS demyelination may have significant implications to management and clinical trials.

## Introduction

73–90% of neuromyelitis optica spectrum disorder (NMOSD) patients diagnosed according to the 2015 International panel on NMO diagnosis have aquaporin-4 antibodies (AQP4-IgG) [1, 2]. It is presumed that at least a proportion of the remaining 10–27% of patients, classified as seronegative NMOSD have another disease specific antibody. Antibodies to myelin oligodendrocyte glycoprotein (MOG-IgG) have been increasingly reported in a variety of CNS neuroinflammatory conditions including patients with phenotypes typical for NMOSD [3]. We aimed to determine the prevalence of MOG-IgG in AQP4-IgG-negative NMOSD.

## Methods

The Walton Centre Neurosciences NHS Trust in Liverpool, United Kingdom, is a tertiary neurology hospital that hosts one of the two national multidisciplinary specialist clinics for patients with NMOSD and non-MS demyelinating disorders as part of the UK NMOSD service. We systematically reviewed all patients seen in this clinic over the last 4 years (after the availability of MOG-IgG testing), including clinical information, MRI, and antibody tests. Both AQP4-IgG and MOG-IgG were tested using a validated live cell-based assay with high specificity (John Radcliffe Hospital, Oxford, UK) [4, 5]. This study was approved by Research Ethics Service, NRES Committee London—Hampstead, Ref. no. 15/LO/1433.

## Results

**Fig. 1:**
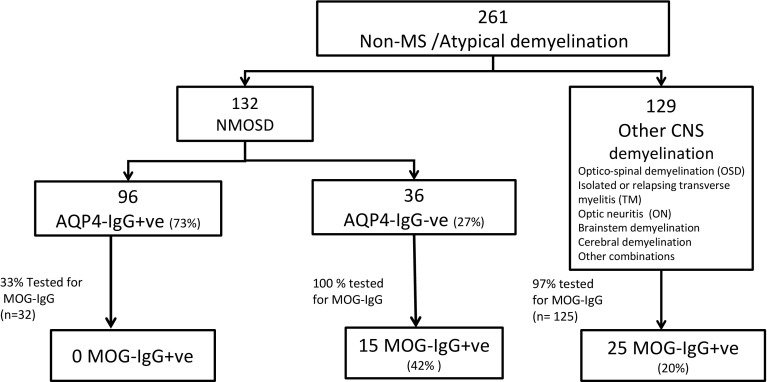
Classification of non-MS/atypical demyelination based on 2015 NMOSD criteria, AQP4-IgG, and MOG-IgG testing. *NMOSD* neuromyelitis optica spectrum disorder, *AQP4 IgG* Antibody to aquaporin 4, *MOG-IgG* antibody to myelin oligodendrocyte glycoprotein, *OSD* optico-spinal demyelination with normal brain MRI

261 unique patients with non-MS/atypical CNS inflammatory conditions attended the clinic and were assessed for NMOSD. All patients were tested for AQP4-IgG. 132 cases satisfied the 2015 NMOSD diagnostic criteria. Of these, 96 (73%) were AQP4-IgG positive and 36 (27%) AQP4-IgG negative. These 36 patients, were tested for MOG-IgG and 15/36 (42%) tested positive. This would account for 11% (15/132) of the total cohort of NMOSD patients (Fig. 1; Table 1). All MOG-IgG-negative patients were Caucasians with a median age of onset of 18 years (8–44 years) and median disease duration of 4.7 years (2–16 years). The predominant clinical phenotype of the demyelinating event was ON (60%), TM (21%), brain (12%), and brainstem (4%).

**Table 1:** Demographic, clinical, and radiological characteristics of the 15 NMOSD patients with MOG-IgG

Patient no.	Age	Sex	Age at onset	Disease duration (years)	Course	Total no. of events	Clinical phenotype (no. of attacks)	First inter-attack interval	Spinal MRI	Baseline brain MRI	CSF oligoclonal bands	EDSS	Current treatment
1	31	F	18	13.4	R	13	ON (13) TM (1)	3 years	LETM	Normal	Negative	4	Subcutaneous IGs (immunoglobulins) and oral prednisolone
2	55	M	44	11	R	7	ON (2) TM (1) brainstem (1) brain syndrome (5)	7 years	Short mid thoracic lesion	Brain stem, cortical and subcortical extensive demy	Positive	3.5	Steroid & mycophenolate
3	31	F	15	16.4	R	2	ON (1) TM (1)	4 years	LETM	Normal	Negative	9	Azathioprine and oral prednisolone
4	21	M	18	2.5	R	5	Brain stem (1) Brain syndrome (1) TM (1) ON (5)	2 months	Multiple short lesions on thoracic cord	Large area of high T2 signal in the posterior brainstem both sides of mid brain	Negative	1.5	Azathioprine switched to rituximab
5	22	M	17	4.7	R	>7	ON (>7) and TM (2)	2 months	LETM	Normal	Unknown	3	Tocilizumab, IVIG six weekly and oral prednisolone
6	30	F	28	2	R	2	ON (1) TM (1)	1 year	LETM	Cerebral ring enhancing lesion supracallosal subcortical	Negative	0	Mycophenolate
7	23	F	8	14.4	R	3	ON (2) TM (2) Brain syndrome (1)	3 years	LETM	Multiple non-specific white matter lesions	Negative	6	Azathioprine and oral prednisolone
8	24	F	17	6.9	R	2	ON (1) TM (1) Brain syndrome (1)	3 months	LETM	Brainstem, left cerebral peduncle, and few non-specific white matter lesions	Negative	1	Azathioprine and oral prednisolone
9	14	F	10	4	R	3	Brain syndrome (1) ON (3) TM (1)	3 month	LETM	Bilateral hemispheric white matter changes	Negative	2.5	Rituximab and mycophenolate
10	28	M	19	8.2	R	4	ON (3) TM (1)	6 years	LETM	Normal	Unknown	4	Mycophenolate
11	44	M	13	31	R	5	ON (3) TM (2)	17 years	LETM	Normal	Negative	3.5	Azathioprine
12	39	F	36	3.1	R	2	Brain stem (1) ON (2)	2.2 years	Normal	Lesion on pons	Negative (161)	3	Mycophenolate and oral prednisolone
13	42	M	38	3.6	R	2	TM (1) Brain stem (1)	2 months	LETM	Peri ependymal pons lesion	Unknown	6	Azathioprine and oral prednisolone
14	28	M	26	2	Single event	1	ON + LETM	Simultaneously	LETM	Normal	Positive	1.5	Mycophenolate
15	45	M	40	5	Single event	1	ON + LETM	Simultaneously	LETM	Normal	Negative	2	None

*F* female, *M* male, *R* relapsing, *ON* optic neuritis, *TM* transverse myelitis, *LETM* longitudinally extensive transverse myelitis, and *IVIG* intravenous immunoglobulins

While we tested all AQP4-IgG-negative patients for MOG-IgG (*n* = 36), only a proportion (33%) of AQP4-IgG-positive patients (*n* = 32) were tested (as double positives are exceptionally rare) (Fig. 1). None were definitely positive. However, one patient was ‘low positive/possibly negative. This patient with one episode of long myelitis also had antinuclear antibodies (1/80 titre with homogenous pattern (nuclear antigens all negative) and was ‘low positive’ for anti-glycine antibodies too. The significance of the MOG-IgG in the context of these additional antibodies is uncertain and may reflect a heightened humoral autoimmune response rather than truly pathogenic dual positivity. This patient has not been included in the MOG cohort in this paper.

We also tested the majority of patients with a demyelinating syndrome referred to the service who did not fulfill the NMOSD criteria (125/129, 97%). Twenty-five (20%) were positive for MOG-IgG. Details of these cases will be the subject of an upcoming separate research paper and are not discussed further here.

We also assessed how many of the MOG-IgG patients with NMOSD phenotype had a relapsing course. Thirteen patients (86%) had a relapsing course. However, a relapsing course was the reason for referral to the clinic in the first place (*n* = 13/13). The median duration of illness for the relapsing patients was 4.7 years (2–16 years). The median inter-attack interval was 1 year (0.16–17) and median EDSS in the relapsing MOG group at last follow-up was 3 (0–9, Table 1). All relapsing patient are on immunosuppressants (Table 1).

We also assessed the proportion of patients with optic neuritis and long myelitis who fulfill Wingerchuk 2006 criteria [6] that are MOG-IgG positive, as this is a clinical question often posed. Of the whole cohort of 261 patients, 75 patients had long myelitis and optic neuritis. Of these 49 were AQP4-IgG positive (66%) and 10 were MOG-IgG positive (13%, or 38% of AQP4-IgG-negative patients) and 16 remained seronegative (21%). Serial testing where done in 14/15 patients (13 relapsing); MOG-IgG was detected in all. Treatment with steroid or immunosuppression does not seem to have an effect on MOG-IgG serostatus in this cohort of predominantly relapsing patients (Table 2).

**Table 2:** MOG-IgG testing in relation to disease course and immunosuppressive treatment. NA: not available

Patient no.	Date of onset	Date of first relapse	Last relapse	Date of start on steroid	Date of start on maintenance immunosuppressive treatment	First-positive MOG-IgG test	Subsequent MOG test year	Titre	Comments
1	Jan 02	May 05	Jul 05	Jan 08	2009	2011	2013, 2014 both positive	NA	Data not clear if was on steroid in first or last relapse, but was on immunosuppressant when tested positive for MOG-IgG
2	2004	2011	2015	2014	2014	2014	2015, 2016, 2017 all positive	300	Patient was not on steroid in first or last relapses, but was on immunosuppressant when tested positive for MOG-IgG after diagnosis and remained positive
3	Jan 99	Apr 03	May 03	Unknown	2003	Apr 14	Jul 14 positive	NA	Data not clear if was on steroid in first or last relapse, but was on immunosuppressant when tested positive for MOG-IgG subsequently
4	Sep 14	Nov 14	May 17	Nov 14	Dec 14	2014	2015 positive	300	Patient was not on steroid in first relapse, but was on steroid and immunosuppressant in last relapse and when MOG-IgG tested and remained positive
							2016 positive	400	
5	Sep 10	Oct 10	Jul 13	At onset	2011	2012	2014, 2015, 2016 all positive	NA	Patient was on reducing dose of steroid in first relapse, and on immunosuppressant and steroid in last relapse and when MOG-IgG was tested and remained positive
6	Aug 13	Sep 14	Sep 14	Sep 14	May 15	Sep 14	2016, 2017 both positive	NA	Patient was not on steroid in first relapse, was on steroid when tested for MOG-IgG initially and in 2016 but off steroid in 2017 and remained positive
7	2001	2004	2010	At onset	2010	2013	2014, 2016 both positive	NA	Patient was not on steroid in first or last relapse, she was on immunosuppressant when tested for MOG-IgG subsequently.
8	Jul 08	Nov 08	Nov 08	At onset	Nov 08	Apr 11	May 11 positive	NA	Data unavailable if patient was on steroid in first relapse, she was on immunosuppressant when tested positive for MOG-IgG
9	Apr 12	Jul 12	Aug 15	At onset	2012	2012	2015, 2016 positive	NA	Patient was on steroid in first relapse and when tested positive for MOG-IgG. She was also positive when was on steroid and immunosuppressant in subsequent relapses.
10	Mar 07	Jul 13	Dec 15	At onset	Jul-14	Apr 14	2016 positive	NA	Patient was not on steroid in first relapse, or first MOG-IgG test. He was on immunosuppressant in last relapse and when remained positive in subsequent testing
11	1984	2001	Mar 13	At onset	2013	2015	No further tests	NA	No available data whether patient was on steroid in first or last relapse, but he was on immunosuppressant when tested positive for MOG-IgG.
12	May 12	Aug 14	Aug 14	At onset	May 15	May 15	2016 positive	NA	Patient was not on steroid in first relapse, but was on steroid when tested positive for MOG-IgG and was on immunosuppressant on subsequent positive test
13	Oct 12	Jan 13	Jan 13	At onset	Aug 13	Jul 13	2014 negative 2015 positive	NA	Patient was on steroid in first relapse, however, immunosuppressant was initiated after MOG-IgG returned positive in 2013, later test one year apart was negative in 2014, and subsequent test in 2015 was positive while still on immunosuppressant
14	Mar 14			At onset	Apr 14	Apr 14	2015, 2016, 2017 all positive	NA	Only one event but patient chose to go on treatment
15	Jun 12			At onset	Not on immunosuppressant	Jun 12	2015 positive	NA	Not on immunosuppression

## Discussion

In a cohort of well-characterised NMOSD patients (*n* = 132), 73% were AQP4-IgG and 11% were MOG-IgG seropositive and 16% remained seronegative. MOG-IgG disease accounts for 42% of the AQP4 IgG-negative seronegative cohort. MOG-IgG was present in 38% of patients with long myelitis and optic neuritis who do not have AQP4 IgG.

86% (13/15) of our patients who satisfy criteria for NMOSD who are MOG-IgG-positive patients have relapsing disease, similar to a recent study [7] who reported that 80% of their MOG-IgG-positive cohort (*n* = 50) followed a relapsing course. However, a relapsing course was the reason for referral to the clinic in the first place (*n* = 13/13) making this a biased sample. Long-term follow-ups of a cohort of MOG-IgG-positive patients after the very first event is required to obtain the true risk of relapse.

Importantly, 20% of patients with non-MS/atypical demyelination who do not satisfy criteria for NMOSD tested positive for MOG-IgG (Fig. 1). Double positive cases (both AQP4-IgG and MOG-IgG) are rare [8–10] with none of the tested patients were definite positives. Since we have tested only 52% (68/132) of the total NMOSD cohort for MOG-IgG, this requires further clarification in future studies.

In conclusion, our study provides the best possible answers at the current time on several questions on the frequency of MOG-IgG patients: NMOSD who are AQP4-IgG negative and MOG-IgG positive (42%), NMO (as per Wingerchuk 2006) with optic neuritis and long myelitis who are AQP4-IgG negative but MOG-IgG (13%). We also found that MOG-IgG is found in 20% of non-NMOSD/non-MS demyelination. It is also estimated that at least 11% of all NMOSD (as per 2015 criteria) is MOG-IgG positive.

Our study has important practical implications. First, the definite diagnosis of MOG-IgG-associated disease offers patients and physicians a better diagnostic label than seronegative NMOSD. Second, as nearly one in every two of seronegative NMOSD, and 1/5 of atypical non-MS demyelination is MOG-Ig positive, testing for these cohorts will be of high yield and worthwhile, compared to testing every demyelination (which in most Caucasian predominant populations is likely to be MS) with attendant costs and risk of false-positive results. Third, it is likely that the long-term disease course and therefore treatment strategies of AQP4-IgG and MOG-IgG is different. If this is the case, MOG-IgG status, should be part of inclusion/exclusion criteria or a variable for stratification in clinical trials. The latter issue may have importance for currently recruiting trials that include seronegative NMOSD.
